# Supplementary material for: Maternal self-efficacy and emotional well-being in Chilean adolescent mothers: the relationship with their children’s social-emotional development
Source: PeerJ. 2022 Apr 12;10:e13162. doi: 10.7717/peerj.13162 (PMC9012175; doi:10.7717/peerj.13162)
Supplement: Supplemental Information 3 [file peerj-10-13162-s003.docx]

**Supplemental Materials**

**S1**. Descriptive summary of ASQ-SE Z scores by sociodemographic factors.

|  | **Self-regulation** | **Adaptive-functioning** | **Affect** | **Social-communication** | **Interaction** | **Social-emotional total score** |
| --- | --- | --- | --- | --- | --- | --- |
|  | **M ± (SD)**  **(Min-Max)** | **M ± (SD)**  **(Min-Max)** | **M ± (SD)**  **(Min-Max)** | **M ± (SD)**  **(Min-Max)** | **M ± (SD)**  **(Min-Max)** | **M ± (SD)**  **(Min-Max)** |
| **Childcare support** |  |  |  |  |  |  |
| Childcare support provided | -.07±.98  (-1.37− 2.54) | .00±1.02  (-1.17− 3.07) | -.11±.96  (-1.37− 1.70) | -.01±.95  (-.87− 3.07) | -.02±.96  (-1.24− 2.56) | -.06±.95  (-1.52− 2.75) |
| Childcare support not provided | .25±.99  (-1.37− 2.29) | -.02±.85  (-1.04− 2.14) | .31±.96  (-1.14− 1.70) | .05±1.15  (-.87− 2.43) | .10±1.09  (-1.24− 3.12) | .22±1.13  (-2.14− 2.59) |
| Statistical test (effect size) | t = 1.17  (d=.32) | t = -.08  (d=.02) | t = 1.63  (d=.44) | t = .25  (d=.06) | t = .46  (d=.13) | t = 1.06  (d=.23) |
| **Mother’s marital status** |  |  |  |  |  |  |
| Single | -.02±1.14  (-1.37− 2.54) | .02±1.03  (-1.17− 3.07) | .05±1.03  (-1.37− 1.70) | .27±1.14  (-.87− 3.07) | -.06±.98  (-1.24− 2.56) | -.06±1.08  (-2.14− 2.75) |
| Boyfriend | .15±.86  (-.96− 1.74) | -.01±1.08  (-1.17− 2.14) | -.26±1.06  (-1.37− 1.69) | -.13±.82  (-.87− 1.61) | -.01±1.03  (-1.24− 2.56) | -.02±.82  (-1.22− 1.64) |
| Co-habiting with a partner | -.11±.88  (-1.37− 2.29) | -.02±.87  (-1.04− 2.14) | .10±.76  (-1.16− 1.70) | -.27±.81  (-.87− 2.43) | .10±.99  (-1.13− 3.12) | -.06±1.02  (-1.52− 2.59) |
| Statistical test (effect size) | F = .41  (η^2^ = .01) | F = .01  (η^2^ = .00) | F = .94  (η^2^ = .02) | F = 2.39  (η^2^ =.05) | F = .18  (η^2^ = .01) | F = .12  (η^2^ =.00) |
| **Mother’s employment** |  |  |  |  |  |  |
| Employed | -.11±.80  (-1.35− 1.38) | .10±.66  (-1.04− 1.12) | .22±1.05  (-1.37− 1.70) | .10±1.02  (-.87− 2.36) | -.12±1.01  (-1.24− 1.89) | -.01±.89  (-1.45− 1.85) |
| Unemployed | .03±1.03  (-1.37− 2.54) | -.02±1.05  (-1.17− 3.07) | -.08±.95  (-1.37− 1.70) | -.02±.98  (-.87− 3.07) | .03±.99  (-1.13− 3.12) | .00±1.02  (-2.14− 2.75) |
| Statistical test (effect size) | t = .49  (d=.15) | t = -.55  (d=.03) | t = -1.09  (d=.30) | t = -.45  (d=.13) | t = .52  (d=.15) | t = .03  (d=.00) |
| **Mother`s level of education** |  |  |  |  |  |  |
| Dropped out of school | .08±.95  (-1.35− 1.93) | -.03±.97  (-1.17− 1.87) | .04±.86  (-1.37− 1.70) | .09±1.12  (-.87− 2.36) | -.07±.90  (-.79− 1.73) | .00±.88  (-1.49− 1.85) |
| Elementary/high school student | -.00±1.10  (-1.37− 2.29) | .21±1.16  (-1.17− 3.07) | .00±1.11  (-1.16− 1.70) | -.05±.73  (-.87− .81) | -.03±.91  (-1.13− 2.56) | .07±.97  (-1.22− 2.15) |
| Completed high school | -.02±.97  (-1.37− 2.54) | -.09±.92  (-1.17− 2.14) | -.04±.95  (-1.37− 1.70) | -.00±1.06  (-.87− 3.07) | .03±1.05  (-1.24− 3.12) | -.03±1.03  (-2.14− 2.75) |
| Statistical test (effect size) | F = .42  (η^2^ = .00) | F = .64  (η^2^ = .02) | F = .04  (η^2^ = .00) | F = .07  (η^2^ =.00) | F = .06  (η^2^ = .00) | F = .07  (η^2^ =.00) |
| **Child gender** |  |  |  |  |  |  |
| Male | -.05±.99  (-1.37− 2.54) | -.00±.99  (-1.17− 2.14) | -.08±1.04  (-1.37− 1.70) | .21±1.07  (-.87− 3.07) | -.10±.88  (-1.24− 2.56) | -.01±1.05  (-2.14− 2.75) |
| Female | -.06±.99  (-1.37− 2.29) | -.00±1.00  (-1.17− 3.07) | .04±.90  (-1.37− 1.70) | -.24±.83  (-.87− 2.36) | .12±1.10  (-1.13− 3.12) | .02±.93  (-1.45− 2.59) |
| Statistical test (effect size) | t = -.47  (d=.11) | t = -.01  (d=.00) | t = -.54  (d=.13) | t = 2.05  (d=.47) ^*^ | t = -.98  (d=.22) | t = -.13  (d=.03) |
| **Preschool center** |  |  |  |  |  |  |
| Attends | -.05±.96  (-1.37− 2.54) | -.11±1.12  (-1.17− 3.07) | -.40±.77  (-1.37− 1.60) | .09±1.09  (-.87− 3.07) | .07±.98  (-1.13− 2.56) | -.08±1.01  (-1.49− 2.75) |
| Does not attend | .03±1.01  (-1.37− 2.29) | .06±.91  (-1.17− 2.14) | .19±1.01  (-1.37− 1.70) | -.05±.93  (-.87− 2.43) | -.04±1.00  (-1.24− 3.12) | .04±.98  (-2.14− 2.59) |
| Statistical test (effect size) | t = .31  (d=.07) | t = .72  (d=.17) | t = 2.70  (d=.66) ^**^ | t = -.58  (d=.14) | t = -.50  (d=.12) | t = .52  (d=.12) |
| **Relatives who live with the child** |  |  |  |  |  |  |
| Mother and father | .41±1.21  (-.96− 2.29) | -.01±.89  (-1.04− 1.34) | .12±.98  (-1.14− 1.69) | -.69±.32  (-.87− .04) | .66±1.52  (-.77− 3.12) | .26±1.50  (-1.52− 2.59) |
| Mother and grandparents | .07±1.01  (-1.37− 2.54) | .02±1.04  (-1.17− 3.07) | -.04±1.07  (-1.37− 1.70) | .11±1.03  (-.87− 3.07) | -.06±.99  (-1.24− 2.56) | .05±.98  (-2.14− 2.75) |
| Mother, father, and grandparents | -.36±.73  (-1.37− 1.38) | -.07±.88  (-1.04− 2.14) | -.02±.57  (-1.16− .75) | -.12±.88  (-.87− 2.43) | -.05±.71  (-1.13− 1.00) | -.26±.80  (-1.22− 2.46) |
| Statistical test (effect size) | F = 1.82  (η^2^ = .05) | F = .06  (η^2^ = .00) | F = .07  (η^2^ = .00) | F = 2.01  (η^2^ =.05) | F = 1.45  (η^2^ = .04) | F = .85  (η^2^ =.02) |

**p* < .05 ***p* < .01

The relationship between each of the factors included in the sociodemographic questionnaire and children’s social-emotional development (ASQ-SE Z scores) was analyzed. The results showed that girls performed significantly better than boys in social-communication (t (77) = 2.05; p = .043 d = 0.47), and children that attended preschool demonstrated better affect (t (69) = 2.92; p = .005 d = 0.66).

**S2**. Multiple mediation model for Maternal self-efficacy on children’s adaptive functioning


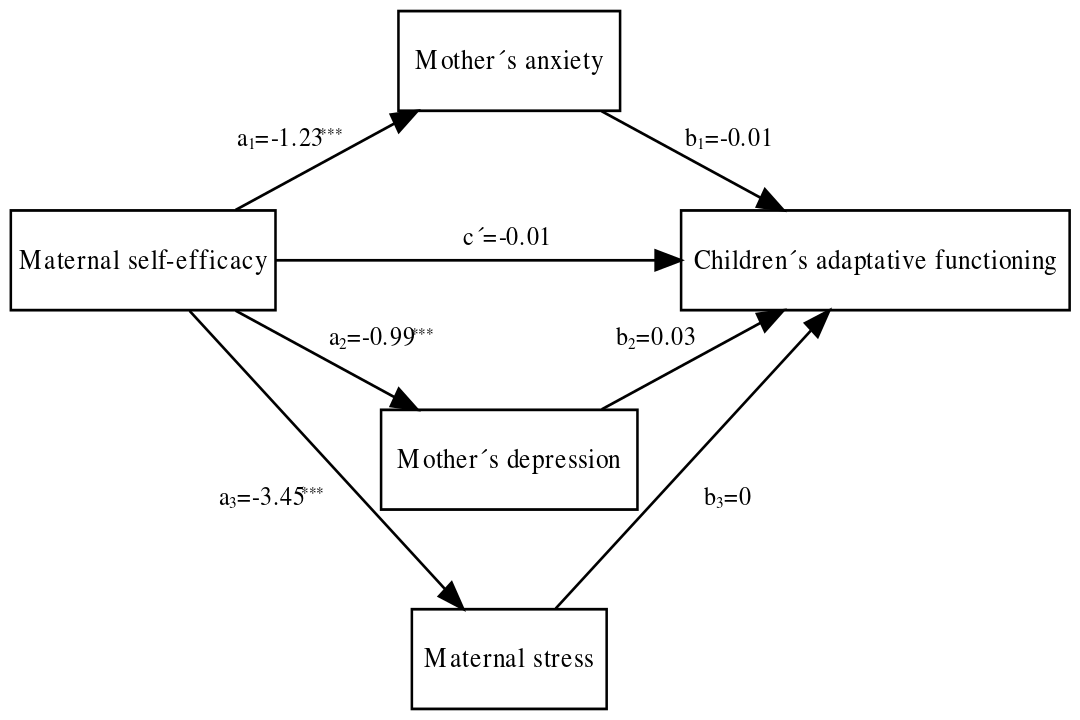


|  | Children’s adaptive functioning | | |
| --- | --- | --- | --- |
|  | Coeff. | SE | *p* |
| Maternal self-efficacy | -.01 | .11 | .91 |
| Mother’s anxiety | -.01 | .04 | .79 |
| Mother’s depression | .03 | .05 | .62 |
| Maternal stress | 0 | .02 | .91 |
| Intercept | -.03 | 1.25 | .98 |
|  | R^2^=.01 | | |
|  | *F* (4, 74) = .14, *p* =.97 | | |

The direct effect between maternal self-efficacy and children’s adaptive functioning was nonsignificant (c’= -0.01, p= .91; 95% CI = [-0.23 – 0.20]). As for the indirect effects on children’s adaptive functioning, neither mediated via mother’s anxiety (a_1_b_1_= 0.01; 95% BCa-CI = [-0.08– 0.12]), via mother’s depression (a_2_b_2_= -0.03; 95% BCa-CI =[-0.12 – 0.06]), nor via maternal stress (a_1_b_1_= -0.01; 95% BCa-CI = [-0.17– 0.14] allowed concluding effects significantly different from zero. Finally, the total effect yielded a non-significant result (c = -0.03; 95% CI = [-0.17– 0.11]). This model presented a low predictive capacity and globally cannot be considered useful for predicting children’s adaptive functioning (R^2^= .01; F (4, 74) = 0.14, p=.97).

**S3**. Multiple mediation model for Maternal self-efficacy on children’s affect


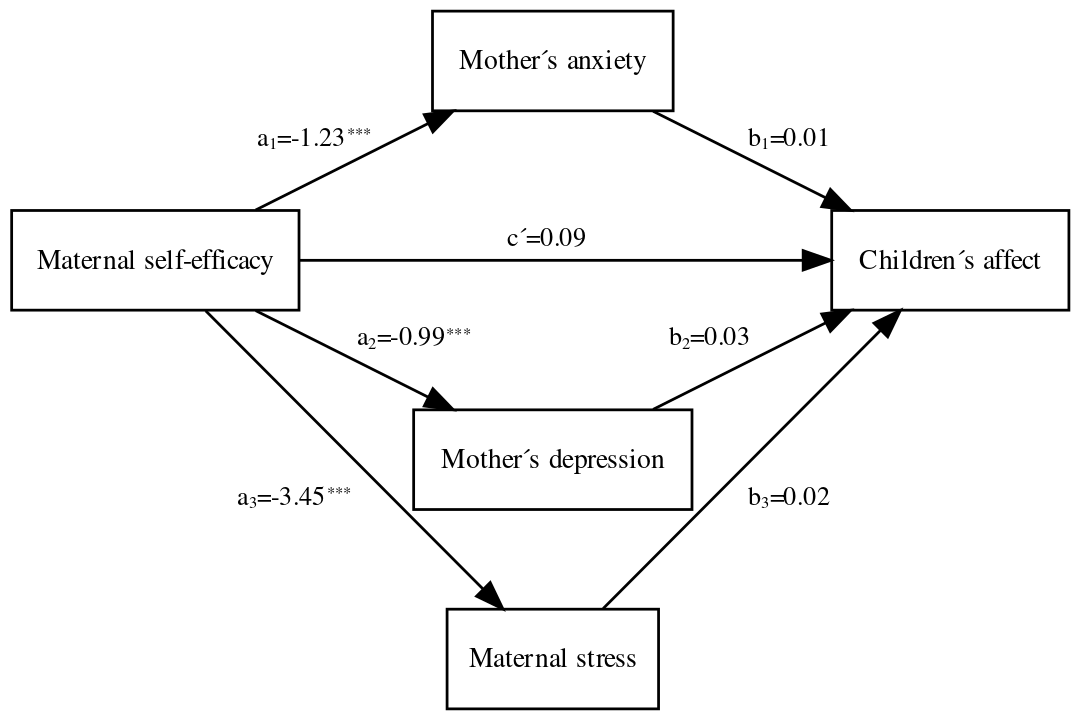


|  | Children’s affect | | |
| --- | --- | --- | --- |
|  | Coeff. | SE | *p* |
| Maternal self-efficacy | .09 | .11 | .38 |
| Mother’s anxiety | .01 | .04 | .86 |
| Mother’s depression | .03 | .05 | .52 |
| Maternal stress | .02 | .02 | .47 |
| Intercept | -1.30 | 1.22 | .29 |
|  | R^2^=.02 | | |
|  | *F* (4, 74) = .45, *p* =.77 | | |

The direct effect between maternal self-efficacy and children’s affect was nonsignificant (c’= 0.09, p= .38; 95% CI = [-0.12 – 0.30]). As for the indirect effects on children’s affect, neither mediated via mother’s anxiety (a_1_b_1_= -0.01; 95% BCa-CI = [-0.12– 0.08]), via mother’s depression (a_2_b_2_= -0.03; 95% BCa-CI = [-0.15 – 0.08]), nor via maternal stress (a_1_b_1_= -0.06; 95% BCa-CI = [-0.23– 0.09] allowed concluding effects significantly different from zero. Finally, the total effect yielded a non-significant result (c = -0.002; 95% CI = [-0.14– 0.14]). This model presented a low predictive capacity and globally cannot be considered useful for predicting children’s affect (R^2^= .02; F (4, 74) = 0.45, p=.77).

**S4**. Multiple mediation model for Maternal self-efficacy on children’s social communication
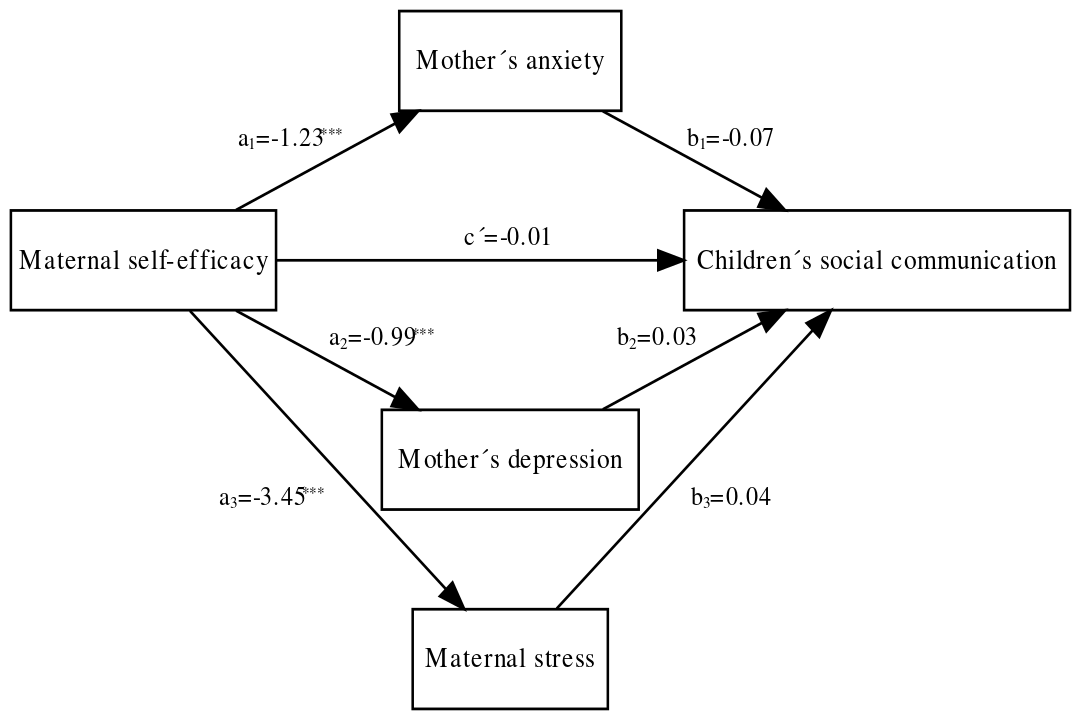


|  | Children’s social communication | | |
| --- | --- | --- | --- |
|  | Coeff. | SE | *p* |
| Maternal self-efficacy | -.01 | .10 | .94 |
| Mother’s anxiety | -.07 | .04 | .06 |
| Mother’s depression | .03 | .05 | .49 |
| Maternal stress | .04 | .02 | .10 |
| Intercept | -.46 | 1.20 | .70 |
|  | R^2^=.10 | | |
|  | *F* (4, 74) = 1.95,*p* =.11 | | |

The direct effect between maternal self-efficacy and children’s social communication was nonsignificant (c’= -0.01, p= .94; 95% CI = [-0.21 – 0.20]). As for the indirect effects on children’s social communication, neither mediated via mother’s anxiety (a_1_b_1_= 0.09; 95% BCa-CI = [0.002– 0.18]), via mother’s depression (a_2_b_2_= -0.03; 95% BCa-CI =[-0.13 – 0.08]), nor via maternal stress (a_1_b_1_= -0.12; 95% BCa-CI = [-0.34– 0.04] allowed concluding effects significantly different from zero. Finally, the total effect yielded a non-significant result (c = -0.08; 95% CI = [-0.22– 0.06]). This model presented a low-medium predictive capacity and globally cannot be considered useful for predicting children’s social communication (R^2^= .10; F (4, 74) = 1.95, p=.11).

**S5**. Multiple mediation model for Maternal self-efficacy on children’s interaction
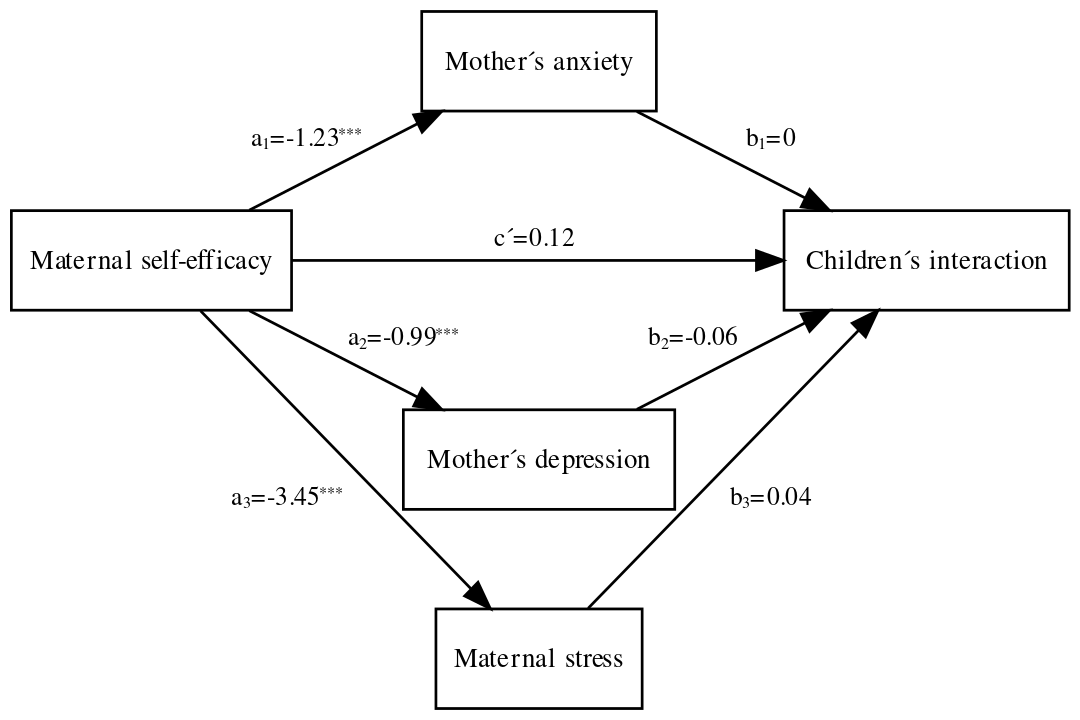


|  | Children’s interaction | | |
| --- | --- | --- | --- |
|  | Coeff. | SE | *p* |
| Maternal self-efficacy | .12 | .11 | .26 |
| Mother’s anxiety | 0 | .04 | .99 |
| Mother’s depression | -.06 | .05 | .26 |
| Maternal stress | .04 | .02 | .07 |
| Intercept | -1.53 | 1.22 | .22 |
|  | R^2^=.05 | | |
|  | *F* (4, 74) = 1.05, *p* =.39 | | |

The direct effect between maternal self-efficacy and children’s interaction was nonsignificant (c’= 0.12, p= .26; 95% CI = [-0.09 – 0.33]). As for the indirect effects on children’s interaction, neither mediated via mother’s anxiety (a_1_b_1_= 0.001; 95% BCa-CI = [-0.10– 0.09]), via mother’s depression (a_2_b_2_= 0.06; 95% BCa-CI =[-0.05 – 0.17]), nor via maternal stress (a_1_b_1_= -0.14; 95% BCa-CI = [-0.30– 0.01] allowed concluding effects significantly different from zero. Finally, the total effect yielded a non-significant result (c = 0.04; 95% CI = [-0.10– 0.18]). This model presented a low predictive capacity and globally cannot be considered useful for predicting children’s interaction (R^2^= .05; F (4, 74) = 1.05, p=.39).

**S6**. Multiple mediation model for Maternal self-efficacy on children’s social-emotional development

|  | Children’s social-emotional development, | | |
| --- | --- | --- | --- |
|  | Coeff. | SE | *p* |
| Maternal self-efficacy | 0 | .10 | 1.0 |
| Mother’s anxiety | 0 | .04 | .98 |
| Mother’s depression | 0 | .05 | .95 |
| Maternal stress | .04 | .02 | .06 |
| Intercept | -0.9 | 1.19 | .46 |
|  | R^2^=.10 | | |
|  | *F* (4, 74) = 2.06, *p* =.09 | | |


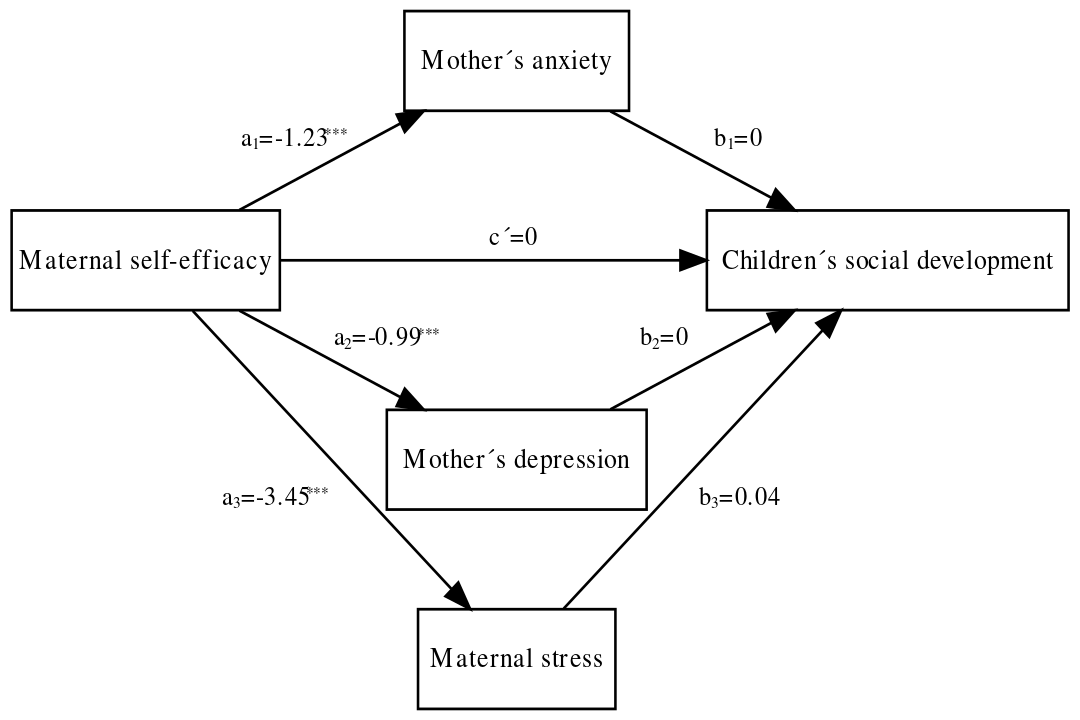


The direct effect between maternal self-efficacy and children’s socioemotional development was nonsignificant (c’= -0.001, p=.10; 95% CI = [-0.21 – 0.21]). As for the indirect effects on children’s socioemotional development, neither mediated via mother’s anxiety (a_1_b_1_= 0.001; 95% BCa-CI = [-0.12– 0.10]), via mother’s depression (a_2_b_2_= 0.003; 95% BCa-CI =[-0.10 – 0.12]), nor via maternal stress (a_1_b_1_= -0.14; 95% BCa-CI = [-0.37– 0.05] allowed concluding effects significantly different from zero. Finally, the total effect yielded a significant result (c = -0.14; 95% CI = [-0.28– -0.0024]). This model presented a low-medium predictive capacity and but globally it cannot be considered useful for predicting children’s socioemotional development (R^2^= .10; F (4, 74) = 2.06, p=.09). Additionally, note that the total effect is contrary to the conjectured one, since a higher mother’s self-efficacy score would be associated with a lower socioemotional development in her child.
